# Developing strategies to address disparities in retention communication during the consent discussion: development of a behavioural intervention

**DOI:** 10.1186/s13063-023-07268-2

**Published:** 2023-04-26

**Authors:** Taylor Coffey, Eilidh Duncan, Heather Morgan, Katie Gillies

**Affiliations:** 1 Health Services Research Unit, Health Sciences Building, University of Aberdeen, Foresterhill, Aberdeen, AB25 2ZD UK; 2 Institute of Applied Health Sciences, University of Aberdeen, Aberdeen, UK

**Keywords:** Intervention development, Clinical trial retention, Behaviour Change Wheel

## Abstract

**Background:**

Clinical trials are essential to evidence-based medicine. Their success relies on recruitment and retention of participants: problems with either can affect validity of results. Past research on improving trials has focused on recruitment, with less on retention, and even less considering retention at the point of recruitment, i.e., what retention-relevant information is shared during consent processes. The behaviour of trial staff communicating this information during consent is likely to contribute to retention. So, developing approaches to mitigate issues in retention at the point of consent is necessary. In this study, we describe the development of a behavioural intervention targeting the communication of information important to retention during the consent process.

**Methods:**

We applied the Theoretical Domains Framework and Behaviour Change Wheel to develop an intervention aimed at changing the retention communication behaviours of trial staff. Building on findings from an interview study to understand the barriers/facilitators to retention communication during consent, we identified behaviour change techniques that could moderate them. These techniques were grouped into potential intervention categories and presented to a co-design group of trial staff and public partners to discuss how they might be packaged into an intervention. An intervention was presented to these same stakeholders and assessed for acceptability through a survey based on the Theoretical Framework of Acceptability.

**Results:**

Twenty-six behaviour change techniques were identified with potential to change communication of retention-information at consent. Six trial stakeholders in the co-design group discussed means for implementing these techniques and agreed the available techniques could be most effective within a series of meetings focussed on best practices for communicating retention at consent. The proposed intervention was deemed acceptable through survey results.

**Conclusion:**

We have developed an intervention aimed at facilitating the communication of retention at informed consent through a behavioural approach. This intervention will be delivered to trial staff and will add to the available strategies for trials to improve retention.

**Supplementary Information:**

The online version contains supplementary material available at 10.1186/s13063-023-07268-2.

## Background

Recruitment and retention to clinical trials remains a challenging issue. A recent review of 151 publicly funded, randomised controlled trials (RCTs) in the UK found that 56% did not meet their required recruitment target within the original timeframe [1]. Such difficulties in meeting recruitment targets then places additional pressures to retain the participants who do enrol [2]. Indeed, certain thresholds of dropout (≥ 20%) pose substantial threats to the generalisability and internal validity of a trial [2–4]. Whilst issues in recruitment and strategies to ameliorate them are widely studied, analogous research on retention appears to be lagging behind [5–7]. For example, the Online Resource for Research in Clinical triAls (ORRCA) website contains 4577 studies on recruitment but only 1338 on retention [8]. This focus on recruitment has been seen as detrimental by trial staff, who feel pressured to prioritise their efforts towards recruitment at the expense of retention [9].

The Cochrane reviews on interventions to improve recruitment and retention identified a number of interventions that have been developed and evaluated with the aim of improving trial conduct [6, 7]. The interventions in these reviews are largely atheoretical, which goes against recommendations on the design of complex interventions—of which most of them would be considered [10]. This is also at odds when considering many of these interventions aim to change peoples’ (either trial staff or trial participants) behaviour in relation to trial recruitment and/or retention [11]. Recruitment and retention both involve many separate but interconnected behaviours and can thus be examined through a behavioural lens. For example, a participant completing and returning a trial questionnaire are two behaviours that are integral to successful retention. Past applications of behavioural science to understand (rather than intervene on) trial recruitment and retention echo the focus on recruitment rather than retention [5]. Further, much of this work has focused on understanding the behaviour of trial participants to understand what drives them to be recruited to, and remain on, a clinical trial [5].

Whilst work to understand the challenges for trial participants is important, it neglects the complex and consistent influence that staff tasked with recruiting and retaining undoubtedly have on participants. In particular, the introduction to the trial through the informed consent process is likely to be a pivotal point for trial staff to engage with potential participants that will affect their likelihood of enrolling but to also lay the groundwork for their continued commitment to the trial [12]. In a recent meta-ethnographic synthesis of reasons for participant dropout, findings suggested that participants who withdrew from trials did so partially due to lack of sufficient information about follow-up and their expectations within the trial being communicated to them during consent [13]. This lack of retention communication during consent is supported by an analysis of recruitment consultations in UK-based RCTs that found no discussion of retention information across 79% of consultations and sparse time allotted in the ones that did [14]. This is particularly a cause for concern when recruitment documents, like participant information leaflets, often fail to mention key aspects that could promote retention, such as the ability to stop or amend treatment and remain in the trial for data collection [15]. For those reasons, facilitation of a fully-informed consent discussion that includes key aspects of information important to retention (e.g., a participant’s expected commitments, the expected impact of their contribution to the trial, etc.) present an attractive means to reconnect the priorities of recruitment and retention into a cohesive best practice.

Through a previously conducted interview study [16], we have identified several important barriers and facilitators to the appropriate dissemination of retention-relevant information at consent by trial staff involved in recruitment. In this paper, we describe the final stages of intervention development, through a systematic behaviour change mapping process and workshop, to co-design intervention content and operationalisation and explore initial feasibility and acceptability of the resulting intervention package meant to facilitate communication of retention information at informed consent.

## Methods

The design of this study has been informed by and leans on established methods using the Theoretical Domains Framework (TDF) and complementary Behaviour Change Wheel (BCW) [17, 18]. These approaches provide a systematic method to diagnose issues within a proposed target behaviour and offer guidance on the available mechanisms to potentially change said target behaviour when designing strategies [17, 18]. Barriers and facilitators to our target behaviour, specified according to the Action, Actor, Context, Target, Time (AACTT) framework [19] (Table 1), have been identified through an earlier piece of work [16] where we interviewed trial staff about their experiences and beliefs about trial retention. The barriers and facilitators generated from those interview findings served as the foundation from which we developed discrete, actionable targets for intervention development. The four steps taken in designing such an intervention are described below.

1. Establishing priority targets for intervention development

**Table 1:** AACTT specification of target behaviour [19]

Action	Actor	Context	Target	Time
Verbal communication about retention to trial (e.g. attendance at clinic, return of questionnaires, if applicable, ability to stop treatment but maintain follow-up)	Trial recruiters	Informed consent discussions	Potential trial participants	Before seeking consent and randomisation

The results of our TDF-based interviews [16] were mapped to the 14 behavioural domains of the TDF [20]. These domains organised the belief statements of our interview participants into distinct categories that represent the primary behavioural constructs believed to underlie our target behaviour. These belief statements were then assessed for their relative impact in influencing the target behaviour so that certain domains were prioritised for intervention. The three criteria that we assessed to establish this impact are frequency and strength of beliefs, along with the presence of conflicting beliefs [18]. From there, we established the relative thresholds of each criterion and any proposed limitations to the number of relevant domains identified through discussion amongst the research team. We excluded some domains at this stage due to the limited practicability of designing a targeted behavioural intervention within the scope of this project. These domains were typically descriptive of behavioural constructs linked to actors or contexts outside the scope of the target behaviour. Nine TDF domains (Knowledge, Skills, Social/professional role and identity, Beliefs about consequences, Reinforcement, Goals, Environmental context and resources, Social influences, and Behavioural regulation) were progressed from the interview analysis to intervention development.

2. Target domains and the identification of behaviour change techniques

Once target domains were established, we then identified the relevant behaviour change techniques (BCTs), suggested by the available evidence, to incorporate into an intervention. BCTs are regarded as the smallest active ingredients within behaviour change interventions [17]. The ability to change behaviour using certain BCTs has been established through past behavioural research, which is available for consultation via the Theory and Techniques Tool [21]. This tool provides evidence of demonstrable links, as well as those inconclusively linked, between each BCT and TDF domain, along with other mechanisms of action [21]. We identified BCTs with available evidence for the relevant domains and agreed which to progress to the next step through discussion guided by the APEASE (Acceptability, Practicability, Effectiveness, Affordability, Scale, and Equity) criteria [17]. Reasons for exclusion were documented in line with one or more of the six APEASE criteria, as applicable. After application of APEASE (with agreement of 3/4 research team members), a refined list of BCTs was generated to progress to a co-design group exercise.

3. Planning the co-design group

A co-design group was planned with trial stakeholders (public partners and trial staff) to further operationalise and discuss potential implementation strategies, along with feasibility and acceptability of the proposed intervention(s). The co-design group was approved by the University of Aberdeen College Ethics Review Board (CERB) (Application No. 2007, Title: CERB/2020/12/2007). The trial staff were invited from a pool of recruiters who participated in our interviews [16] and agreed to be contacted for further involvement in the project. Public partners were invited from public partner groups known to the research team and through solicitation via the Health Service Research Unit’s social media channels. A participant information pack was prepared to introduce co-design members to the aim of the project, our interview findings, and to introduce them to the co-design process and the intervention categories proposed below. This information pack was sent to members the week prior to the group session to allow time to review materials and ask questions in advance. A presentation introducing the aim of the project, our interview findings, and the intervention categories was given at the start of the meeting.

### Conduct of co-design session

The co-design group was conducted virtually via Microsoft Teams, led by a PhD student (TC) and facilitated by the research team (KG, HM, and ED). Participants taking part in the session gave informed verbal consent, which was recorded using the record function in Teams. The recordings were not started until the participant first gave verbal assent to the research team to start that recording. The session ran for two hours and was also recorded in Teams. Each intervention category was introduced by the research team and then the group members were prompted for their feedback. Specific prompts on the intervention categories were adapted from the APEASE criteria [17] to improve accessibility to group members and can be seen in Table 2. Iterative rounds of feedback and discussion were conducted after each question with opportunities to raise conflicting opinions but encouragement to reach agreement. The research team made extensive notes during the discussion and key points were reflected and summarised to identify best practice principles.

4. Intervention acceptability survey

**Table 2:** Intervention acceptability and feasibility prompts, adapted from (Michie et al., 2014)

APEASE criteria	Acceptability	Practicability	Effectiveness	Affordability	Side-effects	Equity
Primary prompt	Do you like it (category), why or why not?	Does it seem practical?	Does it seem effective?	Does it seem affordable?	Can you imagine any unintended effects?	Do you think it could make things more/less fair?
Secondary prompt	Would you find it reasonable if you were asked to use/participate?	Would you be able to use/participate without any major issues?	Would you expect it to work as intended and work well?	Would the proposed changes fit into most budgets?	Could it influence someone to behave in a way they should not?	Could it make someone feel they, or others, were being treated unequally/how they should be treated?

A survey based on the constructs from the Theoretical Framework of Acceptability (TFA), developed to assess acceptability of healthcare interventions, was delivered via Microsoft Forms [22, 23]. As some aspects of the TFA were only relevant to staff members, such as questions regarding participating in an intervention, two versions of the survey were generated. TFA constructs were adapted to questions based on the proposed intervention and the population (i.e., staff members and public partner members). A five-point Likert scale was utilised for each TFA construct question. Additionally, a further optional free text-box was included after each TFA question to allow members to give more detailed explanations to their responses via a free-text option. The surveys distributed to staff members also included questions on the potential operationalisation of certain intervention components. The quantitative data was analysed as frequency counts and free text screened for relevant elaborations of quantitative answers and reflection on potential operationalisation of intervention components. A copy of these surveys is available in Additional file 1.

### Public partner involvement

A public partner (AW) was involved across all stages of this project. This included attending meetings on the initial objective setting for the intervention, participating in discussions during BCT selection, and inputting on the design of the co-design group. The participant information pack and presentation were both reviewed by AW prior to dissemination to ensure accessibility and acceptability.

## Results

The results presented here are reported per the consolidated criteria for reporting qualitative research (COREQ) checklist. This checklist is available in Additional file 2.

### Identification of BCTs

**Fig. 1:**
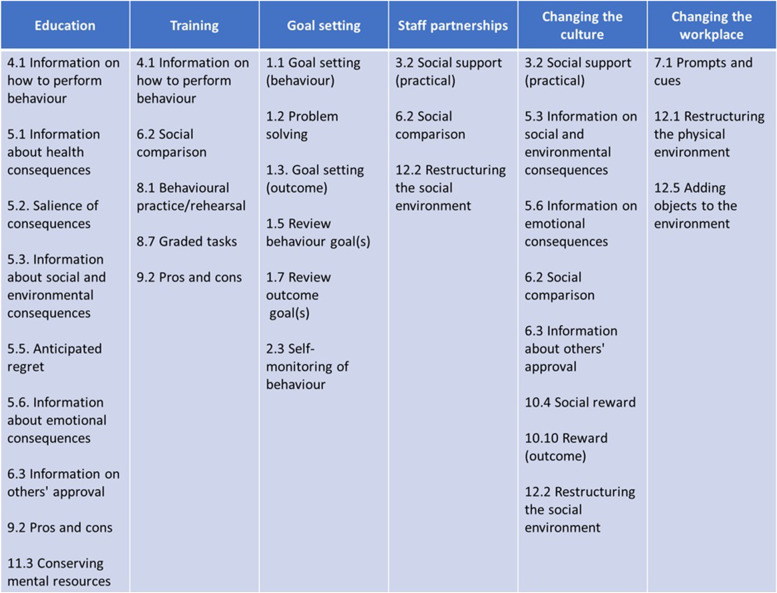
Shortlisted BCTs arranged into potential intervention categories

The research team agreed on a shortlist of 26 BCTs, which can be seen in Table 3 along with the domains that they are known to influence [21]. Related BCTs were grouped under categories that could serve as the foundation for an intervention. We grouped the 26 BCTs from our shortlist into six broad categories based on how they may be used to change our target behaviour. Those six categories (Fig. 1) were as follows: “Education,” “Training,” “Goal setting,” “Staff partnerships,” “Changing the culture,” and “Changing the workplace.”

**Table 3:** Shortlist of BCTs selected through consensus along with the TDF domains they are known to be effective in targeting (within our nine identified domains)

**Behaviour change technique** Definition	**Targeted TDF domain(s)**
**1.1. Goal setting (behaviour)** Set or agree on a goal defined in terms of the behaviour to be achieved	Goals
	Behavioural regulation^a^
**1.2. Problem solving** Analyse, or prompt the person to analyse, factors influencing the behaviour and generate or select strategies that include overcoming barriers and/or increasing facilitators (includes ‘Relapse Prevention’ and ‘Coping Planning’)	Behavioural regulation
	Skills^a^
**1.3. Goal setting (outcome)** Set or agree on a goal defined in terms of a positive outcome of wanted behaviour	Goals
**1.5. Review behaviour goal(s)** Review behaviour goal(s) jointly with the person and consider modifying goal(s) or behaviour change strategy in light of achievement. This may lead to re-setting the same goal, a small change in that goal or setting a new goal instead of (or in addition to) the first, or no change	Goals
**1.7. Review outcome goal(s)** Review outcome goal(s) jointly with the person and consider modifying goal(s) in light of achievement. This may lead to resetting the same goal, a small change in that goal or setting a new goal instead of, or in addition to the first	Goals
**2.3. Self-monitoring of behaviour** Establish a method for the person to monitor and record their behaviour(s) as part of a behaviour change strategy	Behavioural regulation
**3.2. Social support (practical)** Advise on, arrange, or provide practical help (e.g. from friends, relatives, colleagues, ‘buddies’ or staff) for performance of the behaviour	Environmental context and resources
	Social influences
**4.1. Instruction on how to perform behaviour** Advise or agree on how to perform the behaviour (includes ‘Skills training’)	Knowledge
	Skills
**5.1. Information about health consequences** Provide information (e.g. written, verbal, visual) about health consequences of performing the behaviour	Knowledge
	Beliefs about consequences
**5.2. Salience of consequences** Use methods specifically designed to emphasise the consequences of performing the behaviour with the aim of making them more memorable (goes beyond informing about consequences)	Beliefs about consequences
**5.3. Information about social and environmental consequences** Provide information (e.g. written, verbal, visual) about social and environmental consequences of performing the behaviour	Knowledge
	Beliefs about consequences
**5.5. Anticipated regret** Induce or raise awareness of expectations of future regret about performance of the unwanted behaviour	Beliefs about consequences
**5.6. Information about emotional consequences** Provide information (e.g. written, verbal, visual) about emotional consequences of performing the behaviour	Beliefs about consequences
**6.2. Social comparison** Draw attention to others’ performance to allow comparison with the person’s own performance	Social/professional role and identity^a^
	Social influences
**6.3. Information about others’ approval** Provide information about what other people think about the behaviour. The information clarifies whether others will like, approve or disapprove of what the person is doing or will do	Social influences
**7.1. Prompts/cues** Introduce or define environmental or social stimulus with the purpose of prompting or cueing the behaviour. The prompt or cue would normally occur at the time or place of performance	Environmental context and resources
	Beliefs about consequences^a^
**8.1. Behavioural practice/rehearsal** Prompt practice or rehearsal of the performance of the behaviour one or more times in a context or at a time when the performance may not be necessary**,** in order to increase habit and skills	Skills
**8.7. Graded tasks** Set easy-to-perform tasks, making them increasingly difficult, but achievable, until behaviour is performed	Skills
	Goals^a^
**9.2. Pros and cons** Advise the person to identify and compare reasons for wanting (pros) and not wanting to (cons) change the behaviour (includes ‘Decisional balance’)	Beliefs about consequences
**10.3. Non-specific reward** Arrange delivery of a reward if and only if there has been effort and/or progress in performing the behaviour (includes ‘Positive reinforcement’)	Reinforcement
**10.4. Social reward** Arrange verbal or non-verbal reward if and only if there has been effort and/or progress in performing the behaviour (includes ‘Positive reinforcement’)	Reinforcement
	Social influences
**10.10. Reward (outcome)** Arrange for the delivery of a reward if and only if there has been effort and/or progress in achieving the behavioural outcome (includes ‘Positive reinforcement’)	Beliefs about consequences
	Reinforcement
**11.3. Conserving mental resources** Advise on ways of minimising demands on mental resources to facilitate behaviour change	Behavioural regulation
	Environmental context and resources^a^
**12.1. Restructuring the physical environment** Change, or advise to change the physical environment in order to facilitate performance of the wanted behaviour or create barriers to the unwanted behaviour (other than prompts/cues, rewards and punishments)	Environmental context and resources
**12.2. Restructuring the social environment** Change, or advise to change the social environment in order to facilitate performance of the wanted behaviour or create barriers to the unwanted behaviour (other than prompts/cues, rewards and punishments)	Environmental context and resources
	Social influences^a^
**12.5. Adding objects to the environment** Add objects to the environment in order to facilitate performance of the behaviour	Environmental context and resources

^a^Domain-BCT evidence link is inconclusive

### Co-design group findings

#### Co-design contributors

A total of six trial stakeholders (three trial staff and three public partner members) participated in the co-design session. The three trial staff represented three separate trials from our interviews and consisted of two research nurses and one principal investigator/consultant. One of our public partner members was also the project public partner (AW). The two other public partner members were familiar with clinical trials through similar public involvement roles.

### Co-design group feedback

#### Retention training and education

Group members were overall unconvinced by the practicability of an educational and/or training intervention meant to impart best practices for promoting retention. Comments from staff members echoed statements seen in our previous interviews regarding the questionable effectiveness of an intervention that aimed to teach recruiters a particular “script” for how to discuss retention-relevant information during consent. Rather, staff members advocated that recruiters should develop their own tailored approach that is individualised to each consent encounter and participant. The research nurses of the group discussed the interpersonal skills training that they participated in as part of their clinical education and felt the interpersonal aptitudes developed there transferred meaningfully to their research roles. That is, they believed that general training in how to best communicate with patients and respond to their individual needs benefited their abilities to have appropriately personalised consent discussions, including how to discuss retention. These nurses agreed with suggestions that non-clinical research staff could benefit from similar interpersonal skills training.

#### Feedback on retention behaviours

A related topic of discussion within the group involved how trial staff are able to access advice and solicit feedback outside of structured training opportunities. Feedback on their consenting behaviours and their outcomes was typically achieved through engaging with central trial office staff or other trial staff members at regular trial meetings. During these meetings, staff said they were able to reflect on their behaviours within their consent discussions and solicit feedback on whether their techniques were appropriate, as well as seeking advice on how to best handle future conversations. They also shared that seeing specific centres performing well in recruitment and retention prompted them to reach out to these sites to gain insight into how they achieved such notable outcomes, wanting to incorporate these insights into their own processes. The recruiters in the group also mentioned that, whenever possible, they attempted to solicit feedback from patients after consent discussions, particularly if they declined to participate, in order to better understand how their consent behaviours affected participants’ decisions to enrol. Public partner members supported these practices and emphasised their belief that incorporating feedback from participants should be continuous throughout the recruiter’s role to promote adaptive and effective communication behaviours.

#### Promoting engagement in staff and participants

Staff members resonated with findings from the interviews that recruitment can often be an isolated role and that improving connectedness amongst staff could serve several important functions. One research nurse promoted the use of meetings in her past trials and that they became regular opportunities to interact with other trial staff to share recruitment tips and vent frustrations. These meetings also helped to engage staff and foster a greater sense of their contributions to the larger objectives of the trial. In trials without such regular meetings, these opportunities to share advice and connect to other staff, and the trial as a whole, were notably absent.

Public partner members shared examples of engagement strategies from past trial participation that they felt contributed to a positive trial culture experience for participants. Notably, this included the contributions of other staff members outside the immediate research team, such as members of reception staff at study sites and trial-employed taxi drivers, being trained to engage with participants about their trial involvement. Public partners emphasised that this additional engagement outside the primary trial staff could further establish their sense of identity in their role as a trial participant and motivated them to remain in follow-up. The group discussion then considered the utility of trial-wide meetings in promoting retention. In this case, a staff member shared that they had participated in a trial that brought participants together regularly to give them updates on the trial’s progress. These meetings were deemed important in maintaining participant engagement with the trial and solidifying their role as an essential part of the trial’s outcomes. This staff member reflected that very few, if any, of her past trials had a similar level of continued engagement with participants and that it may represent a significant lost opportunity to promote retention.

#### Physical environment

Practical considerations regarding the physical environment in which consent takes place were also discussed. Staff employed similar strategies to those suggested by the research team, which were to have trial documents (e.g. participant information leaflets) in a centralised location for easy access. The hope would be that these documents would help remind staff of aspects of retention that they needed to discuss during their consent discussions. However, staff shared that these types of resources had been largely phased out during COVID to reduce contact surfaces and/or because sites had transitioned to primarily electronic documents. Staff then went on to share their respective recruitment environments and how that has influenced their consent discussions. A dedicated trial space was mentioned as a particularly useful resource, as these dedicated spaces allowed staff to customise their recruitment environment to facilitate consent discussions. One staff member mentioned that the ability to make these spaces more comfortable for participants allowed the conversation to flow more fluidly than it might in a busy or sterile clinic space. This comfortability was believed to allow a more focused conversation about the trial as participants would be more relaxed and attendant to the discussion at hand. Staff would similarly feel less burdened by the pressures of hectic or unfamiliar environments and thus more likely to communicate necessary information about the trial, including retention.

#### Packaging of the intervention

The results of the co-design group necessitated reconsideration and revision of the proposed intervention. A further iteration of a proposed intervention that combined perspectives from the co-design group with BCTs shortlisted from our interview findings was developed. The co-design group gave feedback on this iteration through a survey, the results of which are presented below.

#### Results of the intervention acceptability survey

The acceptability survey was disseminated to group members via email, 12 days from the co-design session. This email included a link to the survey along with a brief description of the rationale for the revised intervention package and an infographic for the intervention. Five of the six members of the group (two staff members and three public partner members) returned surveys (one staff member did not respond to the survey). The average time to complete the survey was 7 min and 50 s.

When considering operationalisation of the proposed intervention, early meetings were agreed to be limited to only one meeting for 1 h. Ongoing meetings were agreed to be at most 1 h. The frequency of these meetings would be quarterly, with the option of reducing later in a trial to only be held when a specific need is identified. Both early and ongoing meetings were agreed to be open to all trial staff, not just recruiters, and would be held either virtually or in-person, or in hybrid settings. As for the other social components of the intervention, participation in the online discussion space would be voluntary, as would being partnered with other trial staff.

Turning to the general impressions on the intervention as a whole, both groups appeared to view the intervention favourably (strongly like or like = 4, no opinion = 1). Staff members did not agree on the perceived amount of effort it would take them to participate in the proposed intervention (no effort at all = 1, a lot of effort = 1). Overall, the group believed the intervention mostly matched their expectations and values (strongly or mostly matches = 4, no opinion = 1). The purpose of the intervention and how it is meant to work were perceived to be clear across the members (completely makes sense = 3, no opinion = 2). The perceived opportunity costs by staff members were generally low (not giving up anything = 1, giving up something = 1). Across group members, the intervention was believed to be able to change the desired behaviour (it is likely to = 2, it definitely will = 1, no opinion = 2). And finally, staff members did not agree in their confidence that they could participate fully in the intervention (unconfident I could = 1, very confident I could = 1).

#### Final intervention package

**Fig. 2:**
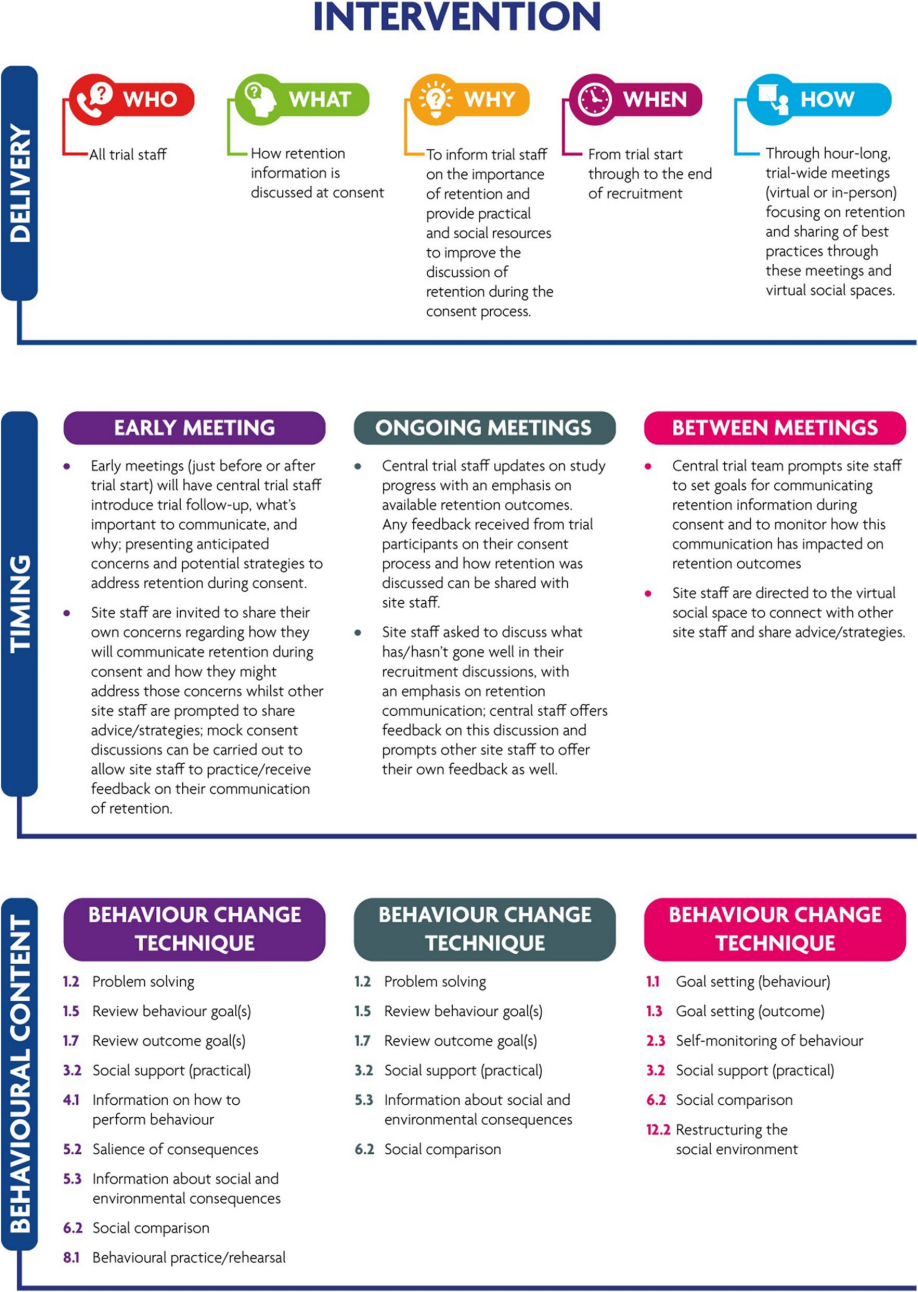
Summary of final intervention package

The intervention package (summarised in Fig. 2) will be delivered through meetings intended to engage trial staff in ongoing education about discussion of retention at the point of informed consent and their role in that process. These meetings will be separated into two types, those conducted early and those conducted throughout the study. The early meetings would be a single session of 1 h and could be held virtually, in-person, or as a hybrid session to facilitate attendance across the trial. Ongoing meetings will be held quarterly, or as need is identified, throughout the timeline of the trial and would similarly be allotted to 1 h with the same flexibilities in delivery. An online social space will be available for staff to seek advice and other feedback, whilst also providing a means to connect with each other and share experiences in retention.

## Discussion

The current study describes the development of an intervention aimed at targeting trial staff’s discussion of retention at the point of informed consent. The results present the process from BCT selection through to measures of acceptability of a proposed intervention. We have taken results from our interview findings with trial recruiters [16], identified key barriers and facilitators that could be targeted to promote effective discussion of retention during consent, identified effective BCTs to target them, and grouped these BCTs into categories of potential interventions. Through our collaboration with stakeholders in a co-design exercise, these categories were refined into a proposed intervention package, one which reconnects the importance of trial retention with the informed consent process.

The findings from the research detailed in this paper will contribute to existing evidence in a number of important ways. Firstly, there is a sparse evidence base of trial conduct interventions targeting trial staff members, with few applying a theoretical approach, and even fewer targeting participant retention [5–7]. A small number of the existing strategies do seek to address issues within both recruitment and retention (e.g. [24–26]). We believe our approach in viewing these recruitment and retention as inextricable from one another is a novel consideration in the design of such strategies, particularly when those strategies are aimed at changing the behaviour of staff members and not participants. Importantly, such an integrated view of the recruitment-to-retention pipeline has potential to shift priorities away from a predominantly recruitment-focused agenda and consider trial participation more holistically. This shift towards an equilibrium in priorities between recruitment and retention will likely prove essential in addressing gaps in adequately informing participants about follow-up and the consequences to retention from such gaps [13–15, 27]. It will also assist in addressing many of the research questions that have been identified through research consensus building exercises with trialists [28, 29].

Examples of staff-focused behavioural interventions were identified in our previous systematic mapping review [5]. Amorrortu et al. [30] utilised Intervention Mapping to design a relationship building strategy to encourage minority-recruiting clinicians to enrol minority participants. Ellis et al. [31] engaged with rural-serving urology practices to understand barriers to discussion of clinical trials and designed an implementation intervention to improve referral rates of these practices to urological cancer trials, utilising the TDF and BCW as their overall approach. Similar work looking to engage maternity healthcare professionals and develop behaviour change interventions aimed at healthcare professionals inviting eligible women to maternity trials is also underway [32].

The intervention was designed with the time constraints of trial staff in mind and has been formatted to reduce the demand on them to adhere to the intervention. The early intervention meetings do so by being a single, 1 h meeting, and the ongoing meetings do so by being quarterly. And participation in the social space will be at the trial staff’s discretion to not create undue burden of participation. In terms of convincing staff about the usefulness of the intervention, the reduced opportunity costs of effective recruitment consultations will be emphasised in several ways. Firstly, in reducing the overall length of conversations through concise communication, also reducing the cognitive demand of lengthy conversations. Secondly, improving the comprehension of trial requirements by the participants, which is both a practical and ethical benefit. And, thirdly, improved retention reducing the amount of overall recruitment conversations that may occur due to those lost to follow-up. Together, these benefits have the potential to leverage the practical considerations of busy trial staff as well as the interpersonal commitments they have to their participants and providing them the best possible care within the trial.

Although we have some preliminary evaluation of feasibility and acceptability of our intervention package, further assessment of these criteria is necessary. As the package is designed to be generalisable, various aspects of its operationalisation are open to refinement through pilot implementation in specific trial contexts. Pilot implementation of the package into ongoing trials can take place via a study within a trial (SWAT) aimed at assessing both feasibility/acceptability and refining intervention components and delivery. This SWAT will likely consist of training of central staff to deliver the intervention by the research team and observations of intervention sessions to evaluate delivery fidelity and other aspects of implementing the intervention. Assessing the effectiveness of the intervention may prove to be challenging, as changes to the target behaviour (communication of retention at consent) may likely be difficult to capture. Instead, selected outcomes of retention (such as retention rates or trial participant satisfaction) as proxies could be used to supplement qualitative implementation outcomes assessed during staff interviews. Ultimately, a randomised control trial of the intervention would be needed to isolate effects of the intervention from various confounds present in naturally occurring differences between sites and trial populations that affect retention outcomes, i.e. cluster randomised. However, if randomisation were to occur at the level of sites, efforts to prevent contamination between sites (i.e. to prevent those sites randomised to receive components of the intervention unintentionally) would be necessary but may prove difficult.

### Strengths and limitations

This study has utilised a systematic, theory-driven behavioural approach to identify appropriate behaviour change techniques to implement within an intervention targeted at recruitment staff. Such a structured approach follows recommendations on complex intervention design [10] and from behavioural scientists to allow transparent dissemination of the proposed mechanisms of actions and their specific behavioural targets [19]. This is essential for evaluation of the intervention’s efficacy, to allow replication/adaptation of the intervention, and to collate evidence of effectiveness across similar contexts and related behaviours [17, 19]. Such replicability was important within our study specifically as the intervention was designed to be applicable to a wide range of trial contexts, rather than being targeted at a specific type of trial or condition. Generalisability was aided by inclusion of recruiters from several distinct UK-based trials in the interviews and co-design process. We have also incorporated multiple perspectives, from trial staff and public partner members, through our co-design process to ensure that both the intervention participants (trial staff) and those affected by the changed behaviour (potential trial participants) were represented in the development process. One limitation of this study is the single co-design session, and its small sample size, utilised due to the time constraints of the PhD project, of which this present study is part. Typically, co-design and other consensus building exercises involve a series of meetings that allow iterative development of the intervention [33, 34]. To mitigate the limitations of our single session, several steps were taken to maximise the time spent discussing the proposed intervention categories (e.g. disseminating an introduction package to participants, inclusion of a public partner throughout all stages of development, and grouping of BCTs to allow focused discussion). A related limitation involves the acceptability survey, which was only sent to members of the co-design group due to the project’s time constraints. The research team opted for a limited sample of respondents to be able to quickly analyse and report some findings on acceptability within the timelines of the project. However, this small sample is likely to limit the generalisability of the intervention’s acceptability and further investigation with wider trial teams and public partners would be necessary to mitigate these limitations.

## Conclusion

Our study has demonstrated the utility of applying a behavioural approach to design an intervention targeted at staff involved in the trial recruitment process to improve trial retention. By reconceptualising the role of recruiters to also include priorities around participant retention, our intervention aims to shift the narrative surrounding trials to a more balanced research culture that does not neglect retention in favour of recruitment. Evaluation of the intervention to determine effectiveness is now needed.

## Supplementary Information

**Additional file 1. **Copy of acceptabilitysurveys. A copy of the online acceptability surveys sent to co-design group participants.The version sent to staff member proceeds the version sent to public partners.

**Additional file 2. **COREQ checklist. Copy ofCOREQ checklist.

## Data Availability

The dataset supporting the conclusions of this article is included within the article (and its additional file). No data is available for sharing beyond those published.

## Abbreviations

AACTT: Action, Actor, Context, Target, Time
APEASE: Acceptability, Practicability, Effectiveness, Affordability, Scale, and Equity
BCTs: Behaviour change techniques
BCW: Behaviour Change Wheel
CERB: College Ethics Review Board
COREQ: Consolidated criteria for reporting qualitative research
ORRCA: Online Resource for Research in Clinical triAls
RCTs: Randomised controlled trials
SWAT: Study within a trial
TDF: Theoretical Domains Framework
TFA: Theoretical Framework of Acceptability
