# Supplementary material for: Developing strategies to address disparities in retention communication during the consent discussion: development of a behavioural intervention
Source: Trials. 2023 Apr 26;24:296. doi: 10.1186/s13063-023-07268-2 (PMC10134580; doi:10.1186/s13063-023-07268-2)
Supplement: Supplementary file 1 — Additional file 1. Copy of acceptabilitysurveys. A copy of the online acceptability surveys sent to co-design group participants.The version sent to staff member proceeds the version sent to public partners. [file 13063_2023_7268_MOESM1_ESM.pdf]

Some questions about our new intervention (for trial staff members)

You will have received a summary of a new type of intervention based on our discussion in the co-design group. If you've not done so, please review that summary before completing the questions below. Please enter in as much information as you can, but at least selecting an answer from the 5-point scale that most closely resembles your opinion. Your answers will be anonymous, so feel free to answer openly.

* Required

Early and ongoing meetings

This first section is going to ask some questions about the early and ongoing meetings to see how they might best be implemented in practice.

1. For early meetings, would one meeting or a series of meetings (2 or more) be better? *

- ◯ One meeting
- ◯ Two or more
- ◯ No early meetings are necessary

2. For early meetings, how long should the meeting(s) be? *

- ◯ Under an hour
- ◯ Between 1 and 2 hours
- ◯ 2 or more hours

3. Do you think these early meetings should just include recruiters or should it be open to all trial staff (e.g., those who are more involved with follow-up)? *

- ◯ Just recruiters
- ◯ All trial staff

4. Would you prefer these early meetings to be in-person or virtual (e.g., over Zoom, Microsoft Teams, etc.)? *

- ◯ In-person
- ◯ Virtual
- ◯ No preference

5. For ongoing meetings, how often would you prefer to have them? *

- ◯ Monthly
- ◯ Quarterly (i.e., every 4 months)
- ◯ Every 6 months
- ◯ Only when a specific need comes up

6. For ongoing meetings, how long should the meetings be? *

- ◯ Under an hour
- ◯ Between 1 and 2 hours
- ◯ 2 or more hours

7. Do you think ongoing meetings should just include recruiters or should it be open to all trial staff (e.g., those who are more involved with follow-up)? *

- ◯ Just recruiters
- ◯ All trial staff

8. Would you prefer these ongoing meetings to be in-person or virtual (e.g., over Zoom, Microsoft Teams, etc.)? *

- ◯ In-person
- ◯ Virtual
- ◯ No preference

Questions about the between meeting components

This second section asks some questions about the proposed additions that occur between meetings.

9. Would you participate in an online space (e.g., a discussion board) with other recruiters to either share tips or ask for advice?

- ◯ Just to share tips
- ◯ Just to ask for advice
- ◯ To both share tips and ask for advice
- ◯ Would not participate

10. Would you volunteer to be partnered with another staff member in the trial?

- ◯ Yes
- ◯ Maybe
- ◯ No

Questions about the overall intervention

The following questions relate to the whole, 3-part intervention package.

11. How do you feel about the intervention?

	Strongly dislike	Dislike	No opinion	Like	Strongly like
Statement 1	◯	◯	◯	◯	◯

12. Can you tell us why you answered that way? For example, is there a particular part of the intervention that you don't like or the intervention as a whole?

13. How much effort do you think it would take to participate in the intervention? *

	No effort at all	A little effort	No opinion	A lot of effort	Huge effort
Statement 1	◯	◯	◯	◯	◯

14. Can you tell us why you answered that way? For example, do some parts seem like they would take more effort than others?

15. Does it match the expectations and/or values you hold for yourself? *

	Does not match	Matches somewhat	No opinion	Mostly matches	Strongly matches
Statement 1	◯	◯	◯	◯	◯

16. Can you tell us why you answered that way? For example, do certain parts not match your values and for a specific reason?

17. Does the purpose of the intervention and how it’s meant to work make sense? *

	Makes no sense	Makes some sense	No opinion	Mostly makes sense	Completely makes sense
Statement 1	◯	◯	◯	◯	◯

18. Can you tell us why you answered that way? For example, are there specific parts of the intervention that don't make sense or is it the intervention as a whole?

19. Do you feel like you would be giving anything up by participating? *

	Not giving up anything	Giving up something	No opinion	Giving up a lot	Giving up everything
Statement 1	◯	◯	◯	◯	◯

20. Can you tell us why you answered that way? For example, do you think you'd be giving up too much time that could be spent doing something else?

21. Do you think the intervention will achieve what it’s meant to (i.e., to help recruiters have better discussions about retention during consent)? *

	It will not	It is somewhat likely to	No opinion	It is likely to	It definitely will
Statement 1	○	○	○	○	○

22. Can you tell us why you answered that way? For example, do some parts seem like they will work, while others may not?

23. Do you feel you’d be able to fully participate in the intervention?

	Very unconfident I could	Unconfident I could	No opinion	Confident I could	Very confident I could
Statement 1	○	○	○	○	○

24. Can you tell us why you answered that way? For example, do you think there are parts that are unnecessary to include?

25. Is there anything else you'd like to say about the intervention that you feel hasn't been addressed?

This content is neither created nor endorsed by Microsoft. The data you submit will be sent to the form owner.

Microsoft Forms

Some questions about our new intervention (for patient-public members)

You will have received a summary of a new type of intervention based on our discussion in the co-design group. If you've not done so, please review that summary before completing the questions below. Please enter in as much information as you can, but at least selecting an answer from the 5-point scale that most closely resembles your opinion. Your answers will be anonymous, so feel free to answer openly.

* Required

1. How do you feel about the intervention? *

	Strongly dislike	Dislike	No opinion	Like	Strongly like
	○	○	○	○	○

2. Can you tell us why you answered that way? For example, is there a particular part of the intervention that you don't like or the intervention as a whole?

3. Does it match the expectations and/or values you hold for yourself/others? *

	Does not match	Matches somewhat	No opinion	Mostly matches	Strongly matches
Statement 1	○	○	○	○	○

4. Can you tell us why you answered that way? For example, do certain parts not match your values and for a specific reason?

5. Does the purpose of the intervention and how it's meant to work make sense? *

	Makes no sense	Makes some sense	No opinion	Mostly makes sense	Completely makes sense
Statement 1	○	○	○	○	○

6. Can you tell us why you answered that way? For example, are there specific parts of the intervention that don't make sense or is it the intervention as a whole?

7. Do you think the intervention will achieve what it’s meant to (i.e., to help recruiters have better discussions about retention during consent)? *

	It will not	It is somewhat likely to	No opinion	It is likely to	It definitely will
Statement 1	○	○	○	○	○

8. Can you tell us why you answered that way? For example, do some parts seem like they will work, while others may not?

9. Is there anything else you'd like to say about the intervention that you feel hasn't been addressed?

This content is neither created nor endorsed by Microsoft. The data you submit will be sent to the form owner.

Microsoft Forms
