# Supplementary material for: Developing strategies to address disparities in retention communication during the consent discussion: development of a behavioural intervention
Source: Trials. 2023 Apr 26;24:296. doi: 10.1186/s13063-023-07268-2 (PMC10134580; doi:10.1186/s13063-023-07268-2)
Supplement: Supplementary file 2 — Additional file 2. COREQ checklist. Copy ofCOREQ checklist. [file 13063_2023_7268_MOESM2_ESM.docx]

**Domain 1: Research team and reflexivity**

| Personal Characteristics |  |  |  |
| --- | --- | --- | --- |
| 1.  Interviewer/facilitator | | Which author/s conducted the interview or focus group? | All authors |
| 2.  Credentials | | What were the researcher's credentials? *E.g. PhD, MD* | TC - M.Sc.; KG, ED, HM – PhD |
| 3.  Occupation | | What was their occupation at the time of the study? | TC – PhD student; KG - Director of the Health Care Assessment Programme and Reader;  ED - Research Fellow and Health Psychologist; HM – Lecturer (Scholarship) |
| 4.  Gender | | Was the researcher male or female? | TC – Male; KG, ED, HM – Female |
| 5.  Experience and training | | What experience or training did the researcher have? | TC – James Lind Alliance-led training on conducting consensus building exercises, training in public engagement activities, experience conducting patient-focused sessions in clinical trials |
| Relationship with participants | |  |  |
| 6.  Relationship established | | Was a relationship established prior to study commencement? | A relationship with one public partner member of the group, AW, was established prior as the project-level public partner. The staff members interviewed were known to TC from a prior interview study. No relationships with the other public partners prior to the group. |
| 7.  Participant knowledge of the interviewer | | What did the participants know about the researcher? e*.g. personal goals, reasons for doing the research* | All participants had contact with TC while being recruited to the group and were introduced to the objectives of the overall project and the purposes of the co-design research. All participants were introduced to all researchers during the co-design session. |
| 8.  Interviewer characteristics | | What characteristics were reported about the interviewer/facilitator? e.g. *Bias, assumptions, reasons and interests in the research topic* | Research team members introduced themselves to members of the co-design group, explaining their past experience and interest in the current research topic. |
| **Domain 2: study design** | | |  |
| Theoretical framework | | |  |
| 9.  Methodological orientation and Theory | | What methodological orientation was stated to underpin the study? *e.g. grounded theory, discourse analysis, ethnography, phenomenology, content analysis* | The study was explained to be underpinned by two related behavioural science approaches, the Theoretical Domains Framework and the Behaviour Change Wheel. Lines 107-145 |
| Participant selection | | |  |
| 10.  Sampling | | How were participants selected? *e.g. purposive, convenience, consecutive, snowball* | Lines 147-154 |
| 11.  Method of approach | | How were participants approached? e*.g. face-to-face, telephone, mail, email* | Lines 151-154 |
| 12.  Sample size | | How many participants were in the study? | Lines 211-213 |
| 13.  Non-participation | | How many people refused to participate or dropped out? Reasons? | Lines 294-295 |
| Setting | | |  |
| 14.  Setting of data collection | | Where was the data collected? e*.g. home, clinic, workplace* | Lines 161-165 |
| 15.  Presence of non-participants | | Was anyone else present besides the participants and researchers? | No |
| 16.  Description of sample | | What are the important characteristics of the sample? *e.g. demographic data, date* | Lines 212-215 |
| Data collection | |  |  |
| 17.  Interview guide | | Were questions, prompts, guides provided by the authors? Was it pilot tested? | Lines 154-159, 165-170, 177-179, 189-191, 447-449 |
| 18.  Repeat interviews | | Were repeat interviews carried out? If yes, how many? | N/A |
| 19.  Audio/visual recording | | Did the research use audio or visual recording to collect the data? | Lines 162-165 |
| 20.  Field notes | | Were field notes made during and/or after the interview or focus group? | Lines 170-171 |
| 21.  Duration | | What was the duration of the interviews or focus group? | Line 165 |
| 22.  Data saturation | | Was data saturation discussed? | No |
| 23.  Transcripts returned | | Were transcripts returned to participants for comment and/or correction? | N/A |
| **Domain 3: analysis and findings** | |  |  |
| Data analysis | |  |  |
| 24.  Number of data coders | | How many data coders coded the data? | N/A |
| 25.  Description of the coding tree | | Did authors provide a description of the coding tree? | N/A |
| 26.  Derivation of themes | | Were themes identified in advance or derived from the data? | N/A |
| 27.  Software | | What software, if applicable, was used to manage the data? | N/A |
| 28.  Participant checking | | Did participants provide feedback on the findings? | Lines 174-185, 289-315 |
| Reporting | |  |  |
| 29.  Quotations presented | | Were participant quotations presented to illustrate the themes / findings? Was each quotation identified? e*.g. participant number* | No |
| 30.  Data and findings consistent | | Was there consistency between the data presented and the findings? | Yes |
| 31.  Clarity of major themes | | Were major themes clearly presented in the findings? | Lines 216-285 |
| 32.  Clarity of minor themes | | Is there a description of diverse cases or discussion of minor themes? | Lines 216-285 |
